# The study protocol for the Head Injury Retrieval Trial (HIRT): a single centre randomised controlled trial of physician prehospital management of severe blunt head injury compared with management by paramedics

**DOI:** 10.1186/1757-7241-21-69

**Published:** 2013-09-14

**Authors:** Alan A Garner, Michael Fearnside, Val Gebski

**Affiliations:** 1 CareFlight Ltd, Westmead, NSW, Australia; 2 Emeritus Consultant Neurosurgery, Westmead Hospital, Sydney, NSW, Australia; 3 Biostatistics and Research Methodology, NHMRC Clinical Trials Centre, University of Sydney, Camperdown, NSW, Australia

**Keywords:** Brain injury, Prehospital, Physician, Paramedic, Intubation

## Abstract

**Background:**

The utility of advanced prehospital interventions for severe blunt traumatic brain injury (BTI) remains controversial. Of all trauma patient subgroups it has been anticipated that this patient group would most benefit from advanced prehospital interventions as hypoxia and hypotension have been demonstrated to be associated with poor outcomes and these factors may be amenable to prehospital intervention. Supporting evidence is largely lacking however. In particular the efficacy of early anaesthesia/muscle relaxant assisted intubation has proved difficult to substantiate.

**Methods:**

This article describes the design and protocol of the Head Injury Retrieval Trial (HIRT) which is a randomised controlled single centre trial of physician prehospital care (delivering advanced interventions such as rapid sequence intubation and blood transfusion) in addition to paramedic care for severe blunt TBI compared with paramedic care alone.

**Results:**

Primary endpoint is Glasgow Outcome Scale score at six months post injury. Issues with trial integrity resulting from drop ins from standard care to the treatment arm as the result of policy changes by the local ambulance system are discussed.

**Conclusion:**

This randomised controlled trial will contribute to the evaluation of the efficacy of advance prehospital interventions in severe blunt TBI.

**Trial Registration:**

ClinicalTrials.gov: NCT00112398

## Background

Secondary prehospital insults such as hypoxia, hypotension and hypercarbia have been demonstrated to be associated with poorer outcomes in severe blunt traumatic brain injury (TBI). Many prehospital care systems use advanced level prehospital care providers in the belief that prevention or early correction of these insults will improve patient outcomes. Outcome improvements associated with such prehospital care systems have been difficult to definitively demonstrate however.

Previous retrospective studies of the prehospital system in Sydney, Australia have suggested an improvement in outcome associated with physician prehospital care for patients with severe injury [1,2] and severe blunt TBI from motor vehicle accidents [3]. The Head Injury Retrieval Trial (HIRT) was designed to confirm these findings for patients with severe blunt TBI.

Sydney has a three tier prehospital system. Basic Life Support (BLS) ambulance officers are able to perform bag-valve-mask ventilation, apply external compression for haemorrhage control, apply splints and provide analgesia via methoxyflurane inhalers. Paramedics are able to perform the same procedures as BLS ambulance officers, but in addition are able to intubate orally without adjuvant neuromuscular blockade or sedative/anaesthetic agents, perform needle chest decompression, establish intravenous lines, administer crystalloid solutions, and a limited range of medications such as morphine and midazolam. Physician staffed prehospital services provide an enhanced range of airway management options including intubation assisted by neuromuscular blockade, anaesthetic/sedative agents, surgical cricothyroidotomy and transtracheal jet ventilation. They provide both needle chest decompression and formal tube thoracostomy, and an enhanced range of vascular access options including venous cut downs, adult intraosseous needles, and central line placement. Units of O-negative packed red blood cells are also routinely carried.

At the time of commencement of the trial in Sydney, paramedics managed ~70-80% of patients with severe head injuries, ~15-25% were managed by BLS ambulance officers when paramedics were not available, and ~3% were managed by physician prehospital teams. The reported rate of intubation of unconscious trauma patients by the various levels of prehospital providers was: 

•BLS ambulance officers, 0%

•Paramedics, 36-43%

•Physicians 100% [1-3].

The proportion of patients treated by each team type resulted in the majority of patients with severe head injuries not being intubated prior to reaching a trauma centre. For example in South Western Sydney from 1995 to 1997 16% of unconscious trauma patients received prehospital intubation [4]. Data on prehospital correction of hypotension is less readily obtainable, but a previous study comparing physicians and paramedics in NSW found a significantly higher rate of correction of hypotension in physician treated patients, (79% in physician treated patients versus 41% in paramedic patients [2]).

There are a number of observational studies that have examined the effectiveness of prehospital treatment by advanced intervention teams in severe TBI [3,5-8] most finding an improvement in outcome, but no previous Randomised Controlled Trial (RCT). A RCT of prehospital treatment strategies for severe TBI is important as the cost to society of the severe neurological impairment that often results is very high, as is the cost of systems of prehospital care that are designed to manage such patients but which currently lack a rigorous evidence base.

## Method and design of the HIRT

### Objective

To determine whether prehospital management of patients with severe blunt TBI by addition of a physician prehospital team to standard paramedic prehospital care results in better Glasgow Outcome Scale (GOS) scores at six months post injury, compared with management by the standard paramedic system alone.

### Design

The study is an investigator-initiated, single centre, prospective, parallel group, randomised, controlled trial conducted in the greater Sydney area of New South Wales, Australia. The trial is registered with Clinicaltrials.gov ID NCT00112398.

### Setting

The study is being conducted in the Sydney coordination area of the Ambulance Service of New South Wales (ASNSW) which has a population of approximately 4.5 million persons. The area is predominantly urban. The physician team is based near the demographic centre of Sydney and is able to access the majority of the Sydney population within 10 minutes helicopter flying time, although the catchment includes areas up to 20 minutes flying time from the base location.

### Case identification, eligibility and participants

The ASNSW Computer Assisted Dispatch (CAD) system is monitored by a member of the physician team for eligible patients via an internet link. The case identification process and its efficacy in identifying severely injured paediatric patients has been described elsewhere [9]. Incidents were randomised if patients met the following criteria:

•Patient identified as an adult (>15 years) by the person placing the emergency call

•Blunt trauma mechanism

•Data collected by the call taker indicated the patient was unconscious or had an altered level of consciousness

If there was doubt about the level of conscious a call back is made to the person who placed the emergency call and the incident was randomised if the patient was unable to obey command. In some cases it is not possible to gain any information about the level of conscious of the patient and randomisation occurs based on high energy mechanism such as fall greater than five metres or pedestrian struck by truck. Incidents are excluded where there was indication that there are five or more casualties as special arrangements outside of the normal system are utilised by the ASNSW to deal with these circumstances.

### Randomisation

Randomisation is via a computerised Interactive Voice Response System (IVRS) provided by the Australian National Health & Medical Research Council (NH&MRC) Clinical Trials Centre. In the event of failure of the IVRS, randomisation is conducted by computer generated random tables produced by the NHMRC Clinical Trials Centre by selection of the next in a sequence of sealed, opaque envelopes containing the assignment to treatment group.

Incidents are stratified into mechanism subgroups (transportation, falls or other mechanism) prior to randomisation. Stratification was performed as a previous study of a very similar system identified a differential treatment effect based on injury mechanism [10].

### Interventions

Standard care is provided to patients regardless of the arm to which the patient is allocated by the randomisation process. If the patient was allocated to the physician team, the team is dispatched to the scene by helicopter in addition to the usual ground paramedic response.

The ambulance dispatch centre supervisor or officers on scene are able to request a physician team response regardless of treatment group allocation, as per the system that existed prior to commencement of the study.

#### Treatment groups

Allocation to standard care

Intervention by road paramedics was according to ASNSW written protocols. Trauma interventions included:

•Cannulation and intravenous crystalloid fluid infusion

•Supraglottic airway devices and bag-valve-mask ventilation

•Intubation without anaesthesia or muscle relaxation

•Needle chest decompression

•Sedation & analgesia

•Midazolam for seizures

•Splinting and spinal immobilisation

•Monitoring of ECG, SaO_2,_ and manual blood pressure

As noted above a physician team could also be responded by the dispatch centre supervisor or at the request of officers on scene. Medical teams contracted to, or provided by the ASNSW, respond in this circumstance rather than the study physician team.

#### Allocation to physician team

The physician team consists of consultant physicians with specialist certification in anaesthesia, emergency medicine or intensive care medicine with a minimum of 12 months prehospital experience, plus an ASNSW paramedic. Interventions in addition to that provided by the paramedic system include:

•Anaesthesia and muscle relaxation to facilitate intubation

•Waveform capnography and a portable ventilator

•Automated non-invasive blood pressure measurement

•Surgical airway

•Tube thoracostomy

•Hypertonic saline

•Packed red blood cells

•Adult intraosseous access

•Portable ultrasound

If the patient is randomised to the intervention arm and the physician team does not arrive with the patient by the time the road paramedic crew is ready to depart, the road crew departs without waiting for the physician team. The only exception to this is cases of airway obstruction, where the team is expected to arrive on the scene in shorter time than it would take to transport that patient to the nearest hospital. Conversely, if the road paramedic crew reaches the patient prior to the physician team and assesses the patient as not requiring physician level intervention they are able to cancel the physician team.

Treatment by the physician team follows standard therapy for the management of severe trauma as de termined by the Royal Australasian College of Surgeons Early Management of Severe Trauma/Advanced Trauma Life Support training program.

GCS scores are recorded by the first road paramedic crew to arrive at the incident scene. Where the physician crew arrived on scene prior to the road paramedics, the GCS score was confirmed with the road paramedics, documented on the patient treatment record and countersigned by the road paramedic.

Regardless of treatment group, patients are trans ported to the nearest major (Level 1) trauma service in accordance with ASNSW transport protocols. Transport is by the fastest available vehicle given the location, traffic and weather conditions. This is typically by road. Helicopter is used only where there is a clear time advantage. After arrival in the receiving trauma centre, the interventional component of the study has concluded and all management beyond that time is according to the standard management policies of the treating trauma centre. The trial design is deliberately pragmatic with no attempt to direct care after patient arrival in the trauma centre with in-hospital care determined by the policies of each institution.

### Follow-up and outcome measures

Surviving, consenting patients who have:

•a GCS of 12 or less at first contact by the treating team

•a fall in GCS to 12 or less unrelated to sedative/anaesthetic medication prior to arrival in the emergency department

•and/or an Abbreviated Injury Scale (AIS) score of 3 or more for the head region (2005 definitions)

Are interviewed by telephone at six months post injury to determine level of functional recovery. All interviews are conducted by a single nurse researcher who is blinded to treatment group. If the patient cannot be contacted at six months, the same nurse researcher assigns a Glasgow Outcome Scale score by reviewing the patient’s medical record at their last contact with the treating hospital.

All other patients identified from the randomised incidents have their survival status at hospital discharge determined. Patients who meet any of the criteria listed below for significant injury also have physiological and anatomical injury severity data plus interventions collected. A functional definition of severe injury is utilised as Injury Severity Scores (ISS) are not routinely available from the receiving trauma hospitals. Neither of these groups have functional assessments performed at six months.

#### Definition of significant injury

•Died at any time prior to hospital discharge

•Admission to High Dependency Unit (HDU) or Intensive Care Unit (ICU)

•Four or more rib fractures

•Insertion of tube thoracostomy/s

•Spinal cord injury with deficit

•Blood transfusion > 4 units packed red blood cells in the first 24 hours

•Required laparotomy, thoracotomy, craniotomy or interventional radiology

•One or more fractured femurs or fractured pelvis requiring fixation / embolisation

•Burns > 20% Body Surface Area or intubation for airway burns

•Fall in GCS of 2 or more points from initial GCS (if not drug induced) in first 4 hours post arrival in ED

•Documented to be in Post Traumatic Amnesia for more than 1 week post injury

**Figure 1:**
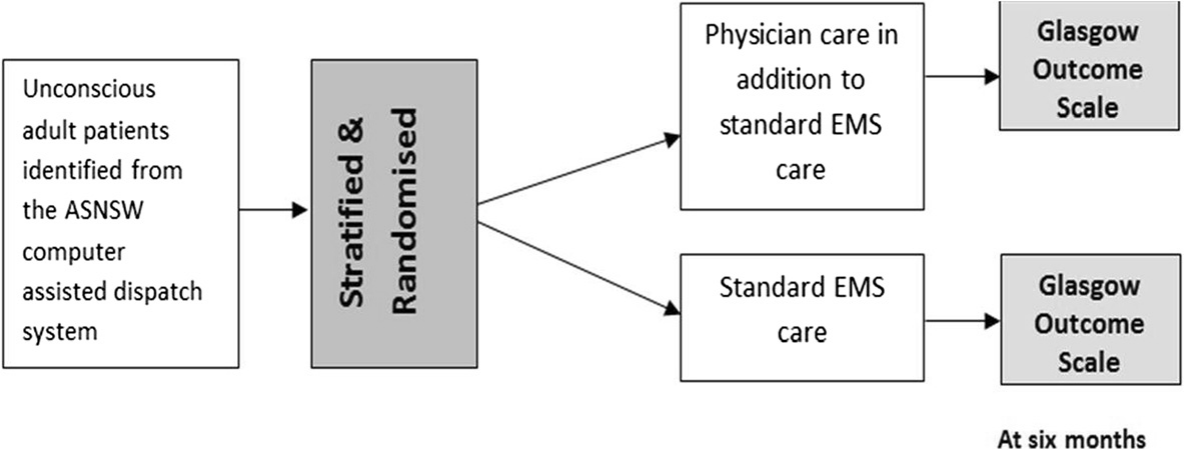
Trial schema.

Figure 1 provides a schema of trial.

### Main outcome measure

•Glasgow Outcome Scale [11] (GOS) at six months post injury. GOS which reflects disability & handicap is the most widely used outcome measure after traumatic brain injury [12], is of particular value in allowing the outcome of different groups of patients to be compared in a simple and easily interpreted fashion [13] and has been recommended as a measure of outcome for clinical trials [14].

### Secondary outcome measures

#### General

•Extended Glasgow Outcome Scale (EGOS) score at six months post injury [12].

•Length of hospital and intensive care unit stays.

•30 day survival and survival to discharge from the acute care hospital.

#### Correction of prehospital secondary insults

All comparisons will be made between first recorded prehospital physiological data and those documented on arrival in the receiving trauma centre.

•Correction of prehospital hypoxia (defined as SpO_2_ <92%).

•Correction of prehospital hypotension (defined as systolic blood pressure < 90 mmHg).

#### Rate of prehospital interventions

•Rate of prehospital intubation

•Volumes and types of fluid administered

•Rate of thoracic decompression

•Other interventions performed

All data analysis is to be on an intention to treat basis. Evaluation of predictors of outcome will be by entering predictor variables into an appropriate logistic regression model with GOS as the dependent variable.

### Subgroup analyses

Due to the nature of the case identification system it is anticipated that many patients randomised will not have severe TBI. Subgroups of patients that have severe and moderate TBI as defined by their initial GCS at the incident scene are therefore the principle outcome groups for the trial. It is anticipated that the greatest treatment effect will be discernable in the patients that meet the severe TBI criteria. Retrospective data from the Sydney system indicated prehospital treatment by a physician team to be significantly associated with a better functional outcome (OR = 2.70, 95% CI: 1.48–4.95) for motor vehicle trauma patients with GCS < 9.

Subgroup analyses will therefore be performed on patients who;

1. Are identified as having a Glasgow Coma Score (GCS) <9 by the team that initially assesses the patient at the scene, and have vital signs present. This group is the primary subgroup for outcome analysis in the trial.

2. Are identified as having a Glasgow Coma Score (GCS) between 9 and 12 by the team that initially assesses the patient at the scene.

Analysis of the above groups by mechanism subgroups will also be conducted as a previous cohort study of a very similar system suggested that the treatment effect would be found almost exclusively in transportation trauma patients [10].

### Computerised Tomography (CT) scans

All cerebral computerised tomography (CT) scans are interpreted by one neurosurgeon investigator (MF) who is blinded to treatment group.

### Sample size calculation

Using the proportions from previously published study of the Sydney system [3], and the 5 point GOS, 48% of patients were classified into categories 4 and 5 under a standard care strategy. A sample size of 510 patients (255 per group) who meet criteria for first subgroup analysis (GCS < 9 on first contact) will have an 80% power to detect an increase of at least 23% into the categories 4 and 5 using the intervention assuming a 5% level of significance and a two-tailed comparison. A 5% rate of non-compliance was assumed as physician care was already provided to a small percentage of patients in the Sydney prehospital care system prior to commencement of the trial.

A secondary endpoint of the study is the proportion of surviving patients who will change status from experiencing a ‘poor’ outcome to a ‘good’ outcome. (“Poor” outcome is defined as either severe disability or persistent vegetative state equivalent to 2 and 3 on the GOS scale whilst a “good” outcome is either moderate disability or good recovery equivalent to a score of 4 or 5). Assuming a rate of 20% in the ‘poor’ outcome category in the control group, the sample size will have 80% power with 95% confidence to detect a decrease in poor outcomes of 9.3% in the intervention arm. If the true rate is 15% then the sample size will have sufficient power to detect a decrease of at least 8%.

If patients who meet the first subgroup analysis criteria withdraw from follow up, it was planned to recruit additional patients until the calculated sample size had been attained.

### Statistical analyses

The primary analysis will compare all randomised patients on the two arms with respect to:

•Proportion of patients in categories 4 and 5 of the GOS scale

•Proportion of patients who are alive and have a ‘poor’ outcome (score of 2 or 3 on the GOS scale).

•Length of stay - in hospital and in intensive care

•30 day survival.

Analysis of proportions will be performed using chi-squared tests (and where appropriate, ‘exact’ tests) whilst a logistic regression analysis and method appropriate for ordinal outcomes will be used to compare proportions adjusting for prognostic factors. Time-to-event analyses (e.g. time to recurrence, overall survival) will be summarised with Kaplan-Meier curves. Exploratory multivariate analyses will be performed using proportional hazards regression methods.

### Interim analyses

Interim analyses will be performed after 140 and 280 patients who meet the criteria for severe head injury (initial GCS < 9) have completed the six-month assessment. Consideration will be given to stopping the study if the p-value for the comparison between the two groups is less than 0.005 after 140 patients and 0.01 after 280 patients have completed their six month assessment.

### Patient safety & ethical considerations

Approval for the trial was granted by the Western Sydney Area Health Service (AHS) Human Research Ethics Committee (HREC). All other Sydney AHS HREC approved data collection and patient follow up for patients treated through the trauma centres in their AHS.

As the target group of the study is unconscious trauma patients consent before enrolment is not possible. The trial however complies with the Australian NH&MRC guidelines for research on unconscious patients who are unable to give consent [15]. The investigators provide, for each surviving subject or their relatives, full written information about the objectives and procedures of the study. They are given the opportunity to ask questions and to decide whether or not they are willing for their results to be included in the dataset. They are also told of their freedom to withdraw from the study at any time and that withdrawal will not affect their future medical care. Subjects included who do not survive will stay in the study; the family will not be asked for consent. This prevents any positive bias in the results.

The study is monitored by an independent data & safety monitoring committee (DSMC) who maintain oversight of trial conduct, patient safety and the interim analyses.

### Study organisation

A study management committee oversees operational conduct of the study. The committee membership comprises:

•Principal investigator, Alan Garner MBBS, MSc, CareFlight.

•Methodology and statistical consultant, A/Prof Val Gebski, NH&MRC Clinical Trials Centre

•Representatives of trial funders (Insurance Australia Group and the NSW Motor Accidents Authority)

•Representative from the ASNSW

•Representative from the NSW Department of Health

•Director of the NSW Institute of Trauma and Injury Management (ITIM)

•Aviation advisor (CareFlight senior pilot)

•An independent chair

### Funding

Funding for the trial was provided by Insurance Australia Group (AUD$11.2 m), CareFlight Ltd (AUD$6 m) and the NSW Motor Accident Authority (AUD$4 m).

### Timeline

The trial commenced recruitment in May 2005. Recruitment was terminated in March 2011.

## Discussion

There has only been a single previous randomised controlled trial (RCT) comparing physician and non-physician teams in prehospital trauma management [16] and no randomised controlled trials evaluating head injury management specifically. The latter group are of particular interest as patients with severe head injuries are possibly the most likely to benefit from advanced on scene interventions which may correct or prevent the effects of secondary insults which have been demonstrated to be associated with poor outcomes.

The study protocol was modified after the first interim analysis as it was realised that a number of patients who met the severe head injury subgroup criteria (initial GCS < 9 at the incident scene) did not have severe head injuries. Some patients who had been initially unconscious rapidly woke up, were subsequently discharged from the emergency department and had no long term sequelae. This problem has been identified in other RCTs of prehospital head injury management [17]. It was also clear that many patients with an initial GCS 9–12 had anatomically severe injury but were excluded from the major analysis. The definition for the principal subgroup was therefore modified to initial scene GCS of 3–12 or GCS falls to ≤12 before hospital arrival without anaesthetic or sedative agents, has vital signs present *and* has an Abbreviated Injury Scale (AIS-2005 definitions) score ≥3. This definition ensures that all patients analysed in the severe head injury subgroup do have severe head injury but at the possible expense of the generalizability of the study as AIS scores are not prospectively available to guide patient triage and management in the prehospital environment. The a priori statistical analysis plan for the trial includes reporting of outcomes for both the initial and modified severe head injury definitions to ensure that a treatment effect in the prospectively identifiable group of patients defined by GCS < 9 alone will not be missed.

It has also been obvious during the recruitment phase of the trial that more patients were dropping into the treatment group from standard care than occurred historically. Two and a half years after commencement of recruitment the ASNSW partially replicated the trial case screening process by implementing a proactive case identification system called the Rapid Launch Trauma Coordinator (RLTC) utilising a paramedic to screen the ASNSW Computed Assisted Dispatch (CAD) system to identify cases appropriate for physician response including in the catchment area of the trial. Drop-ins occurred when the ASNSW dispatch one of their physician teams to patients that are allocated to standard care. Details on the case identification systems and their effect on dispatch to paediatric trauma patients in the Sydney region who were not part of the trial have been reported elsewhere [9].

Due to the higher than anticipated non-compliance rate sensitivity analyses were planned in the a priori statistical analysis in addition to the intention to treat analyses. These were:

•A per protocol analysis in which all non-complying patients would be excluded.

•A treatment received analysis in which all patients would be analysed based on the actual treatment received.

The two planned interim analyses were conducted as planned after follow up of 140 and 280 patients. The first of these analyses was conducted after the follow up of 140 patients who met the initial severe head injury definition and the second analysis was after follow up of 280 patients who met the modified definition of severe head injury. The results were reviewed by the DSMC who recommended that it was safe to continue recruitment.

Recruitment was terminated by the trial management committee in March 2011 after enrolment of 338 patients that met the modified severe head injury subgroup inclusion criteria as:

•Recruitment had continued for nearly two years beyond the planned enrolment period.

•There was increasing pressure from the ASNSW to terminate the trial on the grounds of irrelevance as ASNSW already considered the trial intervention to be standard of care and they therefore considered that they had an ethical duty to provide the treatment to as many trauma patients as possible.

•It was recognised that a significant number of patients were dropping into the treatment group from the standard care group and the policy of ASNSW to dispatch physicians to as many severe trauma patients as possible within the operational area of the trial was likely to exacerbate this issue.

Due to the case identification system that was utilised for the trial [9] a large number of incidents were randomised where no patient was subsequently identified who met the criteria for severe head injury. Many of these patients however met the definition of significant injury used to define patients who were followed up to hospital discharge. Although not the primary outcome of the trial these patients provide an additional randomised group of severe blunt trauma patients for analysis. The a priori statistical analysis plan included survival analyses, comparison of mortality by ISS bands and regression analysis to predict mortality at acute hospital discharge for this patient group. These tests will also be performed for mechanism of injury subgroups.

The results of the study are expected to be published by the first half of 2014.

## Abbreviations

AHS: Area health service; AIS: Abbreviated injury scale; ASNSW: Ambulance service of NSW; BLS: Basic life support; CAD: Computer aided dispatch; CT: Computerised tomography; DRS: Disability rating scale; DSMC: Data and safety monitoring committee; ECG: Electrocardiogram; ED: Emergency department; EGOS: Extended glasgow outcome scale; EMS: Emergency medical service; GCS: Glasgow coma score; GOS: Glasgow outcome scale; HDU: High dependency unit; HIRT: Head Injury retrieval trial; HREC: Human research ethics committee; ICU: Intensive care unit; ISS: Injury severity score; IVRS: Interactive voice response system; NH&MRC: National health & medical research council (Australia); NSW: New South Wales; RCT: Randomised controlled trial; RLTC: Rapid Launch trauma coordinator; TBI: Traumatic brain injury.

## Competing interests

The authors declare that they have no competing interests.

## Authors’ contributions

All authors contributed to the design of the study. AG drafted the manuscript which was reviewed by MF and VG. All authors read and approved the final manuscript.
